# Overweight and Obesity in Adults with Congenital Heart Disease and Heart Failure: Real-World Evidence from the PATHFINDER-CHD Registry †

**DOI:** 10.3390/jcm14134561

**Published:** 2025-06-27

**Authors:** Robert D. Pittrow, Harald Kaemmerer, Annika Freiberger, Stefan Achenbach, Gert Bischoff, Oliver Dewald, Peter Ewert, Anna Engel, Sebastian Freilinger, Jürgen Hörer, Stefan Holdenrieder, Michael Huntgeburth, Ann-Sophie Kaemmerer-Suleiman, Leonard B. Pittrow, Renate Kaulitz, Frank Klawonn, Fritz Mellert, Nicole Nagdyman, Rhoia C. Neidenbach, Wolfgang Schmiedeberg, Benjamin A. Pittrow, Elsa Ury, Fabian von Scheidt, Frank Harig, Mathieu N. Suleiman

**Affiliations:** 1 Department of Cardiac Surgery, University Hospital Erlangen, Friedrich-Alexander-University Erlangen-Nürnberg, 91054 Erlangen, Germany; 2 International Center for Adults with Congenital Heart Disease, Department of Congenital Heart Disease and Paediatric Cardiology, German Heart Center Munich, Technical University Munich, 80636 Munich, Germany; 3 Department of Cardiology, Medizinische Klinik 2—Kardiologie und Angiologie, University Hospital Erlangen, Friedrich-Alexander-University Erlangen-Nürnberg, 91054 Erlangen, Germany; 4 Department of Clinical Nutrition and Preventive Medicine, Hospital Barmherzige Brüder, 80638 Munich, Germany; 5 Chair of Preventive Pediatrics, Department of Sport and Health Sciences, Technical University of Munich, 80638 Munich, Germany; 6 Department of Congenital and Paediatric Heart Surgery, German Heart Center, University Hospital of the Technical University, 80636 Munich, Germany; 7 Division of Congenital and Pediatric Heart Surgery, University Hospital Großhadern of the Ludwig-Maximilians-University, Munich, European Kids Heart Center, 81377 Munich, Germany; 8 Department of Laboratory Medicine, German Heart Center Munich, Technical University Munich, 80636 Munich, Germany; 9 Department of Pediatric Cardiology, Universityhospital Tübingen, 72076 Tübingen, Germany; 10 Helmholtz Centre for Infection Research, Biostatistics, Ostfalia University, 38124 Braunschweig, Germany; 11 Institute for Information Engineering, 38302 Wolfenbüttel, Germany; 12 Department of Sport and Human Movement Science, University of Vienna, 1010 Vienna, Austria

**Keywords:** congenital heart disease, heart failure, obesity, overweight, real-world data, registry

## Abstract

**Background:** The PATHFINDER-CHD Registry is a prospective, multicenter, non-interventional registry across tertiary care centers in Germany. The aim is to analyze real-world data on adults with congenital heart defects (ACHD) or hereditary connective tissue disorders who have manifest heart failure (HF), a history of HF, or are at significant risk of developing HF. This analysis investigates the prevalence and clinical impact of overweight and obesity in this unique population. **Methods:** As of 1st February, 2025, a total of 1490 ACHD had been enrolled. The mean age was 39.4 ± 12.4 years, and 47.9% were female. Patients were categorized according to Perloff’s functional class and the Munich Heart Failure Classification for Congenital Heart Disease (MUC-HF-Class). **Results:** The most common congenital heart disease (CHD) in this cohort was Tetralogy of Fallot, transposition of the great arteries, and congenital aortic valve disease. Marfan syndrome was the most common hereditary connective tissue disease. Of the patients, 46.1% were classified as overweight (32.8%) or obese (13.3%), while 4.8% were underweight. The highest prevalence of overweight (47.1%) was observed among patients who had undergone palliative surgery, whereas untreated patients showed the highest proportion of normal weight (57.2%). Cyanotic patients were predominantly of normal weight. Patients with univentricular circulation exhibited significantly lower rates of overweight and obesity (35%; *p* = 0.001). Overweight and obesity were statistically significantly associated with arterial hypertension, diabetes mellitus, and sleep apnea (all *p* < 0.001). High BMI was linked to increased use of HF-specific medications, including SGLT2 inhibitors (*p* = 0.040), diuretics (*p* = 0.014), and angiotensin receptor blockers (*p* = 0.005). **Conclusions:** The data highlight the clinical relevance of overweight and obesity in ACHD with HF, emphasizing the need for individualized prevention and treatment strategies. The registry serves as a critical foundation for the optimization of long-term care in this population.

## 1. Introduction

The management of adults with congenital heart disease poses an increasing challenge in 21st-century cardiovascular medicine, particularly when complicated by heart failure [1,2,3,4]. Advances in pediatric cardiology, congenital cardiac surgery, and improved long-term care have significantly improved the life expectancy of individuals born with CHD. As a result, the population of ACHD is increasing, currently exceeding 350,000 in Germany and 50 million worldwide [5]. These patients present with a unique clinical profile, characterized by a high burden of sequelae from the underlying CHD as well as associated complications, such as heart failure, pulmonary hypertension, arrhythmias, and non-cardiac comorbidities, including metabolic syndrome and obesity.

The PATHFINDER-CHD Registry (“Patients with Heart Failure Due to Congenital Heart Disease”), a prospective, non-interventional, multicenter cohort study aimed at collecting structured real-world data on ACHD with current or previous HF, or those at risk due to structural or functional abnormalities, was established in 2022. In particular, the registry focuses on the epidemiology, risk factors, and management patterns [6]. PATHFINDER-CHD seeks to address key knowledge gaps by providing robust real-world evidence from specialized tertiary care centers in Germany.

Recent studies highlight the high prevalence of cardiometabolic risk factors in ACHD populations. In a meta-analysis including 110,469 ACHD, 33% had hypertension, 18% were obese, and 7% had diabetes mellitus [7]. In a large cohort of 3905 ACHD, the estimated prevalence of heart failure was 6.4%, with a mean BMI of 25.2 ± 4.9 kg/m^2^ [8].

The role of overweight and obesity in this vulnerable patient group has only rarely been investigated to date [7]. While the relationship between obesity and acquired cardiovascular disease is well documented, little is known about its prevalence and clinical implications in adults with CHD and HF. In adults without congenital heart disease, the co-prevalence of heart failure and obesity is well documented, with obesity contributing significantly to the pathogenesis and progression of heart failure [9]. However, data regarding this association in adults with congenital heart disease remain scarce, despite the growing number of patients reaching adulthood. The unique anatomy and physiology of CHD patients, combined with their lifelong disease burden, require tailored approaches for prevention and management. Obesity may exacerbate symptom burden, reduce functional capacity, and increase the risk of HF and procedural complications. Furthermore, the emergence of new pharmacological treatments in obese patients is often more challenging due to altered drug metabolism and dosing considerations, necessitating individualized therapeutic approaches. Stratification by body mass index (BMI) or body composition is therefore essential to better understand the heterogeneity of comorbidities and treatment patterns within the ACHD cohort [10].

This current cross-sectional analysis specifically examines the prevalence of overweight and obesity in the cohort, by type of surgical status, by ventricular morphology, by functional status, and by cardiac and non-cardiac comorbidities, as well as medication usage across different BMI categories. It aims to identify potential disparities in care and to provide a basis for more personalized, preventive, and therapeutic strategies in ACHD.

## 2. Materials and Methods

The PATHFINDER-CHD Registry is a prospective, multicenter, non-interventional cohort study launched in 2022. It aims to collect structured real-world data on adults (≥18 years) with CHD and either manifest, previous, or preclinical HF. Participating centers are tertiary care institutions across Germany, all certified for their expertise in the management of ACHD [6]. The registry was established with a strong focus on maintaining high-quality standards [11].

The ongoing registry includes data collected between June 2023 and February 2025, with February 2025 serving as the cut-off date for this analysis.

Patients were eligible for inclusion if they were aged 18 years or older, had a confirmed diagnosis of any form of CHD and HF, provided informed consent (either personally or via a legal guardian), and were suitable for long-term follow-up within the registry. The primary exclusion criterion was participation in an interventional clinical trial, as this could compromise the validity of real-world data collection. No additional exclusion criteria were applied in order to reflect the full spectrum of clinical presentations seen in specialized ACHD care. Data collection is performed via a secure, web-based electronic data capture (EDC) system in compliance with the European General Data Protection Regulation (GDPR). All data are pseudonymized and stored in encrypted form. Ethical approval was granted by the leading ethics committee from the Friedrich-Alexander-University Erlangen-Nürnberg (Ref: 23-460-Bn) and the Technical University of Munich (Ref: 2022–582-S-KH), with additional further local approvals obtained as required.

The registry captures both mandatory core data and optional extended variables at baseline and follow-up visits. Documented variables include demographics, type and complexity of CHD, prior surgical or interventional procedures, ventricular morphology, functional classification (Perloff and modified ACC/AHA) [2,12], comorbidities, medication use, as well as weight and height to calculate BMI.

Overweight and obesity are categorized according to the World Health Organization classification: normal weight (BMI 18.5–24.9 kg/m^2^), overweight (BMI 25.0–29.9 kg/m^2^), and obesity (BMI ≥ 30.0 kg/m^2^) [13]. Heart failure was classified using the Perloff’s functional class and the ACC/AHA Heart Failure Classification (2022) modified for CHD. Comorbidities such as arterial hypertension, diabetes mellitus, and sleep apnea were documented based on physician diagnosis and standard clinical criteria as routinely applied in participating certified ACHD centers. Surgical status, ventricular morphology, and types of congenital heart disease were recorded in accordance with standard cardiological practice.

The study complies with the principles of Good Pharmacoepidemiology Practice (GPP) [14] and Good Practice in Secondary Data Analysis (GPS) [15]. All statistical evaluations were conducted on pseudonymized, non-personal data. Descriptive and inferential statistical analyses were performed to identify patterns and associations. Methods included frequency analyses, cross-tabulations, and one-sided chi-squared tests for categorical variables, with statistical significance defined as *p* < 0.05. No imputation or statistical adjustments were applied; missing values were reported as observed. All analyses were performed using SPSS version 29.0 (IBM Corp., Armonk, NY, USA). Longitudinal analyses are planned for future registry follow-up data.

## 3. Results

### 3.1. Patient Characteristics

**Figure 1:**
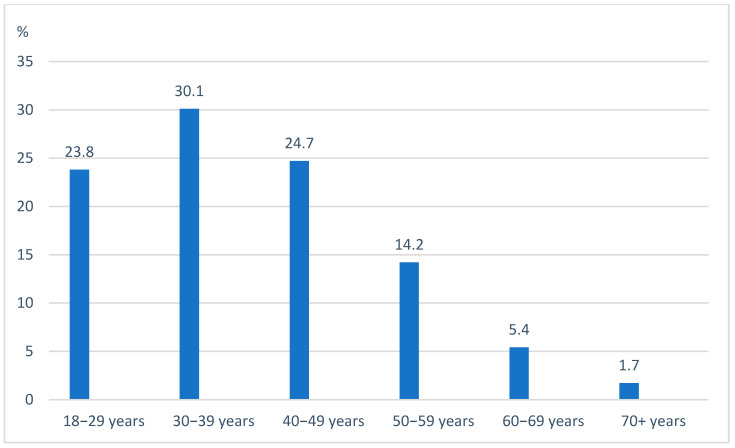
Age by decades.

**Figure 2:**
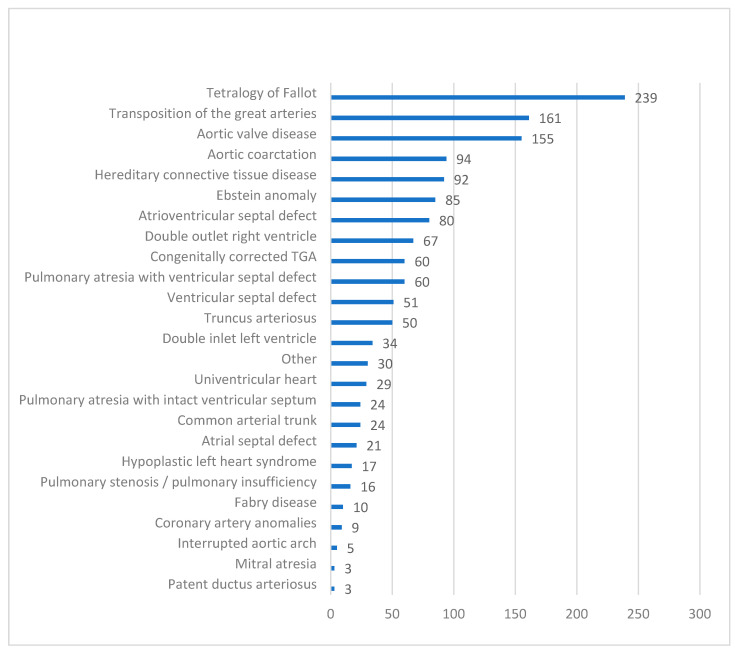
Types of congenital heart defects. Bars show the number of patients.

As of 1 February 2025, 1490 ACHD patients with HF were enrolled in the PATHFINDER-CHD Registry. The mean age was 39.4 ± 12.4 years (range: 18–84), with most patients being in the 4th decade (Figure 1). There were 47.9% female patients. A breakdown by the type of CHD is provided in Figure 2. Of the patients, 33.4% had right-sided heart anomalies, followed by 24.7% with complex CHD, 19.5% with left-sided heart anomalies, and 12.8% with septal defects and vascular malformations (Table 1).

### 3.2. Primary Cyanotic vs. Acyanotic Defects

Acyanotic CHD (n = 651; 45.9%) was slightly less frequent than primary cyanotic CHD (n = 768; 45.9% vs. 54.1%). Acyanotic patients were slightly older (mean 40.9 vs. 38.2 years) and more often female (49.5% vs. 46.9%).

### 3.3. Functional Status

According to Perloff’s functional classification, 91.5% of patients were in functional class (FC) I or II, indicating preserved daily function. Further, 8.2% were in FC III and 0.3% in FC IV. The modified Munich HF classification placed 0.4% in Class A (at risk), 29.7% in Class B (pre-HF), 68.8% in Class C (symptomatic HF), and 1.1% in Class D (advanced HF).

### 3.4. BMI Distribution

A total of 188 patients (13.3%) were classified as obese, highlighting a substantial burden of excess weight in this population. Additionally, 466 patients (32.8%) were overweight, 765 (53.9%) had normal weight, and 71 were underweight or cachectic.

### 3.5. BMI and Treatment Status

Across all treatment groups (treatment-naive, surgically repaired, and interventionally treated), overweight and obesity were prevalent (Table 2). Notably, patients who had undergone palliative surgery exhibited the highest proportion of overweight individuals (47.1%).

### 3.6. Systemic Ventricular Morphology

Of the patients, 79.8% exhibited a left-systemic ventricle, while 9.9% had a right-systemic ventricle and 10.3% a univentricular anatomy. Weight distribution varied by the pathologic anatomy of the systemic ventricle. Patients with morphologic right or left systemic ventricles showed higher rates of overweight and obesity, while patients with univentricular circulation had the highest rate of normal weight (Table 3).

### 3.7. Cardiac Comorbidities

Cardiac comorbidities were frequent, with aortopathies (manifest or at risk) being the most common, followed by arterial hypertension, arrhythmias, and pulmonary hypertension/Eisenmenger syndrome. Arterial hypertension was statistically significantly (*p* < 0.001) associated with overweight/obesity (Table 4).

### 3.8. Non-Cardiac Comorbidities

Non-cardiac comorbidities included in declining order are kidney disease, hypothyroidism, hyperuricemia, liver disease, hyperlipidaemia, neurological disorders, and anemia. A strong association was observed between overweight/obesity and sleep apnea syndrome, as well as with diabetes mellitus (both *p* < 0.001) (Table 5).

### 3.9. Pharmacotherapy

A total of 206 patients were not receiving HF-specific medication. In the remaining cohort, overweight or obese patients were treated with angiotensin II receptor blockers (AT_1_ blockers; *p* = 0.005), diuretics (*p* = 0.014), and SGLT2 inhibitors (*p* = 0.040). Additionally, anticoagulants—such as vitamin K antagonists, non-vitamin K oral anticoagulants (NOACs), and acetylsalicylic acid (ASA)—were more common in overweight/obese patients (Table 6).

## 4. Discussion

The PATHFINDER-CHD Registry was established in response to a notable gap in the scientific and clinical discourse: while obesity is firmly established as a major cardiovascular risk factor in general cardiology, its relevance in the context of adult congenital heart disease has only recently garnered scientific attention. A targeted literature review confirms that this topic has been more intensively addressed within the last decade, with overall limited published evidence to date [16]. Reported prevalence rates for obesity in the ACHD populations vary substantially, ranging from 7% to 26%, while rates of overweight status lie between 22% and 53% depending on study design, patient cohort, and geographic region [7,16,17]. There are no data on obesity in ACHD with different stages of HF.

In contrast to this emerging epidemiological data, current ACHD guidelines still reflect a surprising lack of engagement with the issue. Neither the 2018 AHA/ACC Guideline for the Management of Adults With Congenital Heart Disease [2] nor the 2020 ESC Guidelines [3] include specific recommendations on the assessment, monitoring, or management of obesity in ACHD patients. At best, obesity is mentioned in passing, without substantive discussion of its implications for pathophysiology, prognosis, or therapy.

This neglect is particularly striking when compared to general HF guidelines, where obesity is consistently recognized as a key modifiable risk factor that influences disease onset, progression, and treatment outcomes [18,19]. In contrast, ACHD care has largely overlooked this issue, likely due to the outdated perception that congenital heart defects are primarily pediatric conditions and that adult survivors form a small, exceptional group. Yet this perspective no longer holds: more than 350,000 ACHD patients live in Germany, and an estimated 50 million worldwide [5], with most surviving into adulthood and accumulating additional comorbidities over time.

Studies from Europe and North America consistently show that ACHD—especially those with simple or moderately complex defects—face a comparable or even elevated burden of obesity and associated cardiometabolic risk factors, including hypertension, dyslipidemia, and diabetes mellitus [20,21,22]. Yet, lifestyle counselling and structured weight management are rarely incorporated into their routine follow-up. In a large multicenter study from the German National Register for Congenital Heart Defects, over 50% of ACHD were classified as overweight or obese, while targeted preventive measures were inconsistently applied [23].

The findings from the PATHFINDER-CHD Registry corroborate these observations in a systematically documented, real-world cohort. Nearly half of the included ACHD with HF were overweight or obese (46.1%), with overweight alone affecting one-third of the cohort. This prevalence is consistent with prior international reports [17,24] and highlights the need for structured obesity screening and intervention as part of ACHD care.

Importantly, in PATHFINDER-CHD, BMI was significantly associated with cardiovascular and metabolic comorbidities such as arterial hypertension, obstructive sleep apnea, and diabetes mellitus—conditions known to aggravate HF and compromise long-term outcomes [7,25].

Pharmacological treatment patterns varied significantly with BMI. Overweight and obese patients were more frequently treated with SGLT2 inhibitors, diuretics, and AT blockers, suggesting a greater therapeutic need and/or proactive clinical management in these patients. However, whether these prescribing patterns translate into improved clinical outcomes remains to be determined and warrants further investigation.

An interesting finding of the present analysis is the lower prevalence of overweight and obesity among patients with cyanotic CHD, who were more frequently of normal weight. This observation may reflect underlying pathophysiological differences, including altered energy expenditure, chronic hypoxemia, or reduced nutritional intake in this subgroup [26]. Similarly, patients with univentricular circulation demonstrated the lowest rates of excess weight, further supporting the notion that anatomical and hemodynamic characteristics may significantly influence metabolic phenotypes in ACHD.

While these cross-sectional associations are noteworthy, their clinical relevance remains to be fully elucidated. It is currently unclear to what extent variations in BMI or body composition impact long-term outcomes such as hospitalization rates, exercise capacity, or mortality in this heterogeneous patient population. To address these questions, prospective longitudinal studies are essential.

## 5. Limitations

While the PATHFINDER-CHD Registry offers rich data from ACHD-certified tertiary care centers, the generalizability of its findings may be limited to this setting. Patients treated in high-volume, specialized ACHD centers may not reflect the broader population managed in general or less specialized settings. Additionally, the registry lacks direct measurement of body composition; reliance on BMI alone may underestimate adiposity, particularly in patients with altered body habitus, such as those with connective tissue disorders [25]. Future analyses will benefit from including alternative anthropometric indices, such as waist circumference, waist-to-height ratio, or bioelectrical impedance analysis (BIA) [27], which may offer improved risk stratification [10,28].

Furthermore, the cross-sectional design of the current analysis precludes causal inferences. Longitudinal data are necessary to better understand the impact of weight status on clinical outcomes.

Despite these limitations, this study represents a crucial step toward understanding the burden of obesity in ACHD with HF and offers a foundation for developing targeted interventions to improve long-term outcomes.

## 6. Conclusions

The PATHFINDER-CHD Registry reveals a high prevalence of overweight and obesity among ACHD and HF. These findings underscore the complex interplay between anatomical, functional, and metabolic factors in this vulnerable patient population. Elevated BMI was significantly associated with a range of cardiac and non-cardiac comorbidities and influenced treatment patterns, suggesting a substantial burden on healthcare systems and individual prognosis.

Given the limited evidence base in this field, our data provide an important foundation for future interventional studies, for example, those investigating pharmacological therapies now available to support weight loss. They also support the urgent need for individualized preventive strategies, lifestyle interventions, and multidisciplinary care models that address weight-related issues in ACHD. The implementation of structured weight management should become a standard component of ACHD follow-up, particularly in patients with additional risk factors for cardiovascular deterioration.

The underrepresentation of obesity in current clinical guidelines and the scarcity of dedicated interventions reflect a broader deficiency in integrating cardiometabolic risk management into congenital cardiology. Addressing this gap requires both a paradigm shift in clinical care and a stronger scientific focus on the intersection between CHD, obesity, and heart failure in adult populations. The present study contributes to this emerging field and highlights the urgent need for updated clinical guidelines, targeted research, and individualized therapeutic strategies. Prophylactic measures to avoid obesity should be implemented much earlier in the patient’s life course.

In sum, PATHFINDER-CHD offers critical insights into the real-world health profiles of ACHD with HF and should stimulate the development of new standards of care that incorporate metabolic health as a central therapeutic goal.

## Figures and Tables

**Table 1:** Type and demographics of CHD.

CHD Classification	Frequency n (%)	Mean Age ± SD [Range] (Years)	Female (%)
Complex CHD	351 (24.7%)	37.8 ± 11.4 (18–82)	40.7
Right-sided heart anomalies	474 (33.4%)	40.7 ± 12.9 (18–84)	54.6
Left-sided heart anomalies	276 (19.5%)	39.9 ± 12.6 (19–77)	35.1
Septal defects and vascular malformations	181 (12.8%)	38.8 ± 12.7 (19–79)	60.2
Other CHD	137 (9.7%)	39.1 ± 11.7 (19–76)	51.8
Total	1419 (100.0%)	39.4 ± 12.4 (18–84)	47.9

CHD = congenital heart disease; SD = standard deviation; Right-sided heart anomalies: Tetralogy of Fallot, Ebstein’s anomaly, Pulmonary atresia, Pulmonary valve disease; Left-sided heart anomalies: Aortic valve disease, Aortic stenosis, Coarctation of the Aorta, Interrupted Aortic Arch, Hypoplastic Left Heart Syndrome, Mitral Stenosis; Complex heart anomalies: Transposition of the Great Arteries, Double Outlet Right Ventricle, Double Inlet Left Ventricle, Congenitally Corrected Transposition of the Great Arteries, Univentricular Heart; Septal defects and vascular malformations: Atrioventricular Septal Defect, Ventricular Septal Defect, Atrial Septal Defect, Truncus Arteriosus Communis, Patent Ductus Arteriosus.

**Table 2:** Surgical treatment status.

Treatment Status	Total n	Normal Weight [BMI 18.5–24.9]	Overweight [BMI 25–30]	Obesity [BMI 30+]	*p*-Value
Palliative Surgery	17	7 (41.2%)	8 (47.1%)	2 (11.7%)	0.678
Reparative Surgery	988	540 (54.6%)	316 (32.0%)	132 (13.4%)	0.351
Interventional Treatment	269	135 (50.1%)	98 (36.4%)	36 (13.5%)	0.926
Native	145	83 (57.2%)	44 (30.3%)	18 (12.5%)	0.165
Total	1419	765 (53.9%)	466 (32.8%)	188 (13.3%)	-

n = number of patients. BMI = Body Mass Index.

**Table 3:** Systemic ventricle morphology.

Systemic Ventricle Morphology	Total n	Normal Weight [BMI 18.5–24.9]	Overweight [BMI 25–30]	Obesity [BMI 30+]	*p*-Value
Right-systemic ventricle	140	63 (45.0%)	60 (42.9%)	17 (12.1%)	0.781
Left-systemic ventricle	1133	607 (53.6%)	367 (32.4%)	159 (14.0%)	0.404
Univentricular	146	95 (65.1%)	39 (26.7%)	12 (8.2%)	0.001 *
Total	1419	765 (53.9%)	466 (32.8%)	188 (13.3%)	-

* Statistically significant (*p* < 0.05). n = number of patients. BMI = Body Mass Index.

**Table 4:** Cardiac comorbidities.

Cardiac Comorbidities	Total n	Normal Weight [BMI 18.5–24.9]	Overweight [BMI 25–30]	Obesity [BMI 30+]	*p*-Value
Aortopathy (manifest or at risk)	754	412 (54.6%)	253 (33.6%)	89 (11.8%)	0.348
Art. Hypertension	129	46 (35.7%)	48 (37.2%)	35 (27.1%)	0.001 *
Cardiac arrhythmias	139	73 (52.5%)	41 (29.5%)	25 (18.0%)	0.171
Infective endocarditis	20	11 (55.0%)	6 (30.0%)	3 (15.0%)	0.878
Coronary artery disease	3	1 (33.3%)	2 (66.6%)	-	0.993
Pulmonary hypertension/Eisenmenger	24	7 (29.2%)	10 (41.6%)	7 (29.2%)	0.25
Cyanosis	23	16 (69.6%)	4 (17.4%)	3 (13.0%)	0.207
Other	56	33 (58.9%)	14 (25.0%)	9 (16.1%)	0.157

* Statistically significant (*p* < 0.05). n = number of patients. BMI = Body Mass Index.

**Table 5:** Non-Cardiac comorbidities.

Non-Cardiac Comorbidities	Total n	Normal Weight [BMI 18.5–24.9]	Overweight [BMI 25–30]	Obesity [BMI 30+]	*p*-Value
Anemia	44	24 (54.5%)	18 (40.9%)	2 (4.6%)	0.096
Liver disease/failure	61	27 (44.3%)	26 (42.6%)	8 (13.1%)	0.835
Pulmonary disease	33	20 (60.6%)	8 (24.2%)	5 (15.2%)	0.351
Renal disease/failure	69	33 (47.8%)	25 (36.3%)	11 (15.9%)	0.201
Neurologic disease	46	23 (50.0%)	20 (43.5%)	3 (6.5%)	0.328
Cerebrovasc. accidents	17	7 (41.2%)	8 (47.1%)	2 (11.8%)	0.601
Sleep apnea syndrome	12	2 (16.7%)	6 (50.0%)	4 (33.3%)	<0.001 *
Diabetes mellitus	20	2 (10.0%)	11 (55.0%)	7 (35.0%)	<0.001 *
Hyperlipidemia	59	29 (49.2%)	17 (28.8%)	13 (22.0%)	0.163
Lp(a) increase	18	8 (44.4%)	5 (27.8%)	5 (27.8%)	0.587
Hyperuricemia	66	31 (47.0%)	25 (37.9%)	10 (15.2%)	0.39
Hypothyreosis	67	36 (53.7%)	24 (35.8%)	7 (10.5%)	0.602
Hyperthyreosis	9	1 (11.1%)	6 (66.7%)	2 (22.2%)	0.07

* Statistically significant (*p* < 0.05). n = number of patients. BMI = Body Mass Index. Lp(a) = Lipoprotein a.

**Table 6:** Medications.

Medication	Total n	Normal Weight [BMI 18.5–24.9]	Overweight [BMI 25–30]	Obesity [BMI 30+]	*p*-Value
None	206	124 (60.2%)	64 (31.1%)	18 (8.7%)	<0.001 *
Beta blocker	248	128 (51.6%)	91 (36.7%)	29 (11.7%)	0.773
ACE inhibitor	59	24 (40.7%)	25 (42.4%)	10 (16.9%)	0.113
AT blocker	50	19 (38.0%)	19 (38.0%)	12 (24.0%)	0.005 *
Neprilysin-Sacubitril	31	13 (41.9%)	13 (41.9%)	5 (16.2%)	0.290
Diuretics	108	50 (46.3%)	32 (29.6%)	26 (24.1%)	0.014 *
Aldosterone antagonists	102	52 (51.0%)	30 (29.4%)	20 (19.6%)	0.120
SGLT2 inhibitors	53	23 (43.4%)	18 (34.0%)	12 (22.6%)	0.040 *
Digitalis glycosides	19	12 (63.2%)	7 (36.8%)	-	0.150
Levosimendan	1	1 (100.0%)	-	-	-
Antiarrhythmics	15	9 (60.0%)	6 (40.0%)	-	0.251
Iron, oral	14	6 (42.9%)	8 (57.1%)	-	0.694
PDE-5 inhibitors	22	10 (45.5%)	11 (50.0%)	1 (4.5%)	0.958
ERA	18	8 (44.4%)	8 (44.4%)	2 (11.1%)	0.927
sGC stimulators	2	-	1 (50.0%)	1 (50.0%)	0.249
PRA	1	1 (100.0%)	-	-	-
Sotatercept	0	-	-	-	-
CCB	15	7 (46.7%)	3 (20.0%)	5 (33.3%)	0.287
VKA	153	85 (55.6%)	57 (37.2%)	11 (7.2%)	0.037 *
DOAC/NOAC	64	27 (42.2%)	27 (42.2%)	10 (15.6%)	0.039 *
ASA	49	21 (42.9%)	16 (32.7%)	12 (24.5%)	0.041 *
Clopidogrel	5	2 (40.0%)	1 (20.0%)	2 (40.0%)	0.096
Thyroid hormone therapy	99	49 (49.5%)	34 (34.3%)	16 (16.2%)	0.121

ASA = acetylsalicylic acid; CCB = calcium channel blockers; DOAC = direct oral anticoagulant; NOAC = non-vitamin K oral anticoagulant; ERA = endothelin receptor antagonists; PRA = prostacyclin receptor agonists; VKA = vitamin K antagonist; PDE-5 = phosphodiesterase type 5; sGC = soluble guanylate cyclase; SGLT2 = sodium-glucose co-transporter 2; AT_1_ blocker = angiotensin II receptor type 1 blocker; n = number of patients; BMI = Body Mass Index. * Statistically significant (*p* < 0.05).

## Data Availability

Researchers or clinical centers interested in accessing the dataset for collaborative analyses are encouraged to contact the principal investigators. Data sharing will be considered in accordance with applicable data protection regulations and study governance.

## Abbreviations

The following abbreviations are used in this manuscript:

ACHD	Adult Congenital Heart Disease
ASA	Acetylsalicylic Acid
AT1 blockers	Angiotensin II Receptor Type 1 Blockers
AHA	American Heart Association
ACC	American College of Cardiology
BMI	Body Mass Index
CHD	Congenital Heart Disease
EDC	Electronic Data Capture
ESC	European Society of Cardiology
FC	Functional Class (Perloff Classification)
GDPR	General Data Protection Regulation
GPP	Good Pharmacoepidemiology Practice
GPS	Good Practice in Secondary Data Analysis
HF	Heart Failure
MUC-HF-Class	Munich Heart Failure Classification for Congenital Heart Disease
NOACs	Non-Vitamin K Oral Anticoagulants
PATHFINDER-CHD	Patients with Heart Failure Due to Congenital Heart Disease (Registry)
SD	Standard Deviation
SGLT2 inhibitors	Sodium-Glucose Cotransporter 2 Inhibitors
